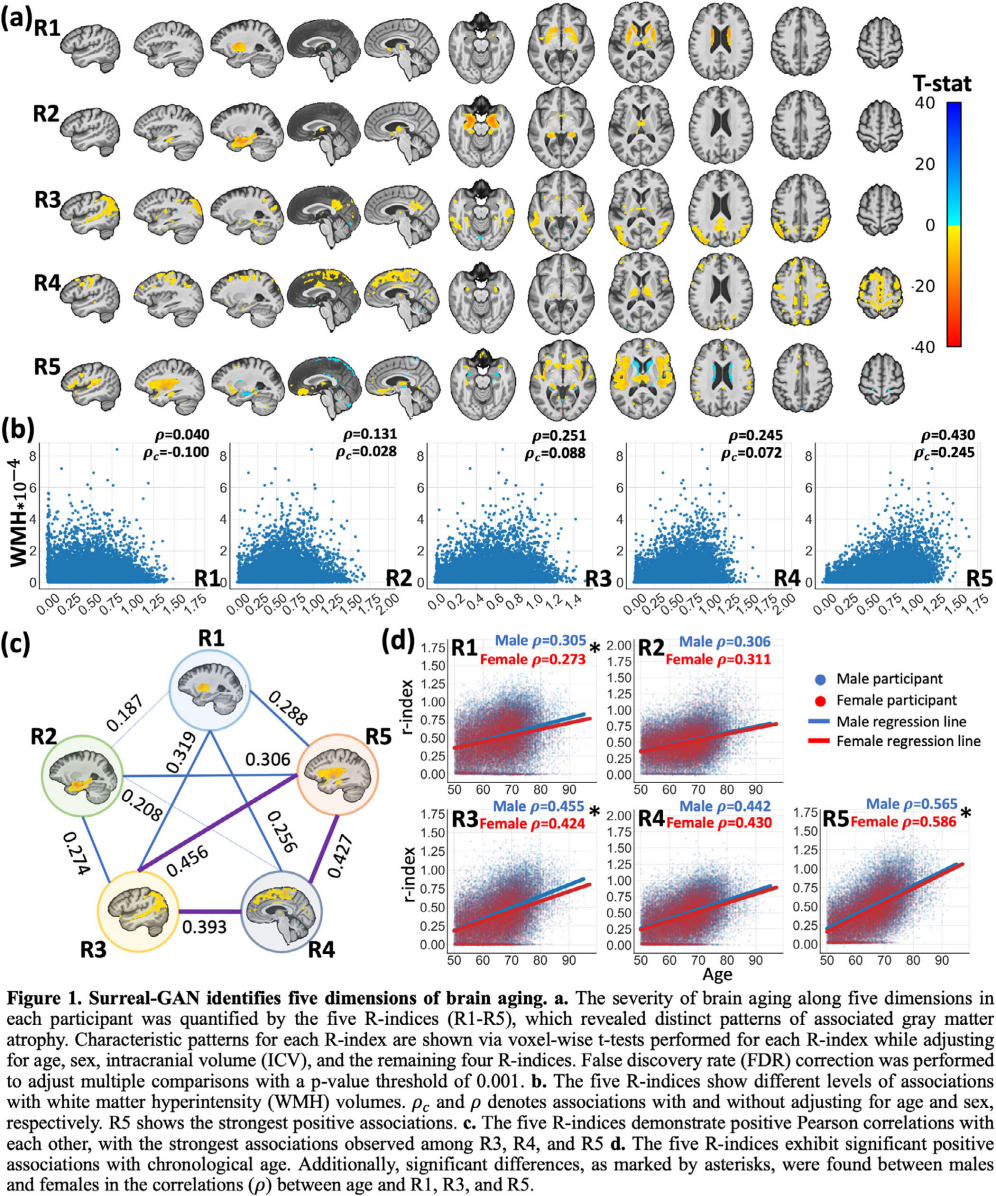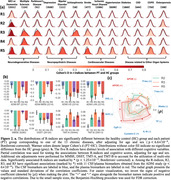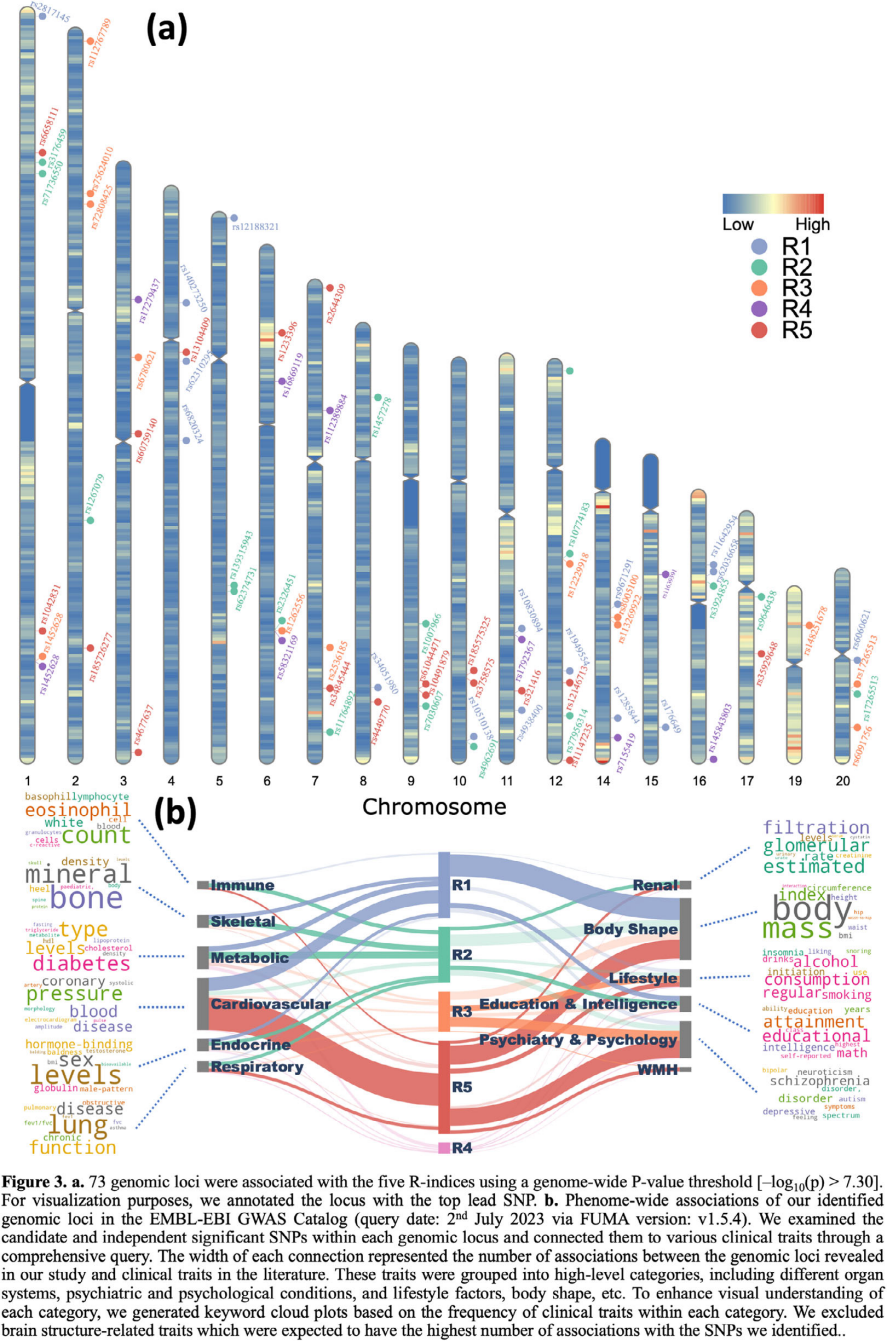# Identifying Five Dominant Dimensions of Neurodegeneration in Brain Aging through Deep Learning: Correlations with Clinical and Genetic Measures

**DOI:** 10.1002/alz.086039

**Published:** 2025-01-09

**Authors:** Zhijian Yang, Junhao Wen, Guray Erus, Christos Davatzikos, the iSTAGING consortium

**Affiliations:** ^1^ Artificial Intelligence in Biomedical Imaging Laboratory (AIBIL), Center for and Data Science for Integrated Diagnostics (AI2D), Perelman School of Medicine, University of Pennsylvania, Philadephia, PA USA; ^2^ University of Southern California, LA, CA USA; ^3^ Artificial Intelligence in Biomedical Imaging Laboratory (AIBIL), Center for and Data Science for Integrated Diagnostics (AI2D), Perelman School of Medicine, University of Pennsylvania, Philadelphia, PA USA

## Abstract

**Background:**

Brain aging is a complex process influenced by various genetic, lifestyle, and environmental factors, as well as by age‐related and often co‐existing pathologies. MRI and AI methods have been instrumental in understanding neuroanatomical changes that occur during aging in large and diverse populations.

**Method:**

We leveraged Surreal‐GAN, a state‐of‐the‐art deep representation learning method, to elucidate the heterogeneity of brain aging by deriving dominant dimensions (patterns) of brain changes and examining their associations with various clinical and genetic factors. Our analysis utilized structural MRIs, clinical variables, and imputed genotype data from a diverse cohort of 49,482 individuals across 11 studies.

**Result:**

Surreal‐GAN identifies five dominant dimensions of neurodegeneration in brain aging: R1, subcortical atrophy; R2, MTL atrophy; R3, parieto‐temporal atrophy; R4: diffuse cortical atrophy; and R5: perisylvian atrophy (Figure 1a). Individuals express varying but non‐exclusive levels of these patterns, quantified by respective R‐indices. The five R‐indices demonstrate positive correlations with each other, with R5 showing the strongest associations with white matter lesion volumes (WMH) and chronological age (Figure 1bc). Clinically, MCI/dementia participants exhibit significantly advanced R‐indices, particularly in R2, R3, and R5. Schizophrenia and Parkinson’s disease participants demonstrate severe aging effects in R3. R5 is associated with neuropsychiatric diseases and other chronic conditions (Figure 2a). Among the dementia‐associated dimensions, R2 displays stronger associations with memory impairment, while R3 is more closely associated with executive dysfunction (Figure 2b). In contrast to R2 and R3, R5 reveals weaker associations with amyloid and tau, but stronger associations with various other CSF biomarkers related to hemostatic and inflammatory mechanisms (Figure 2c). Genetically, GWAS identifies 73 genomic loci significantly associated with R1‐5 (Figure 3a). Previously identified SNPs correlate with various relevant clinical traits, including R5 loci that show associations with cardiovascular and neuropsychiatric conditions and WMH (Figure 3b). Additionally, the R1 dimension correlates with a gene set linked to the response to cortisol.

**Conclusion:**

This study characterized the neuroanatomical heterogeneity related to the aging process by offering a 5‐dimensional representation system with five distinct brain signatures. It elucidates connections to demographics, pathology, and genetics, offering potential for personalized diagnostics, improved patient management, and enhanced precision in clinical trials.